# Gadoxetic Acid-Enhanced MRI-Based Radiomics Signature: A Potential Imaging Biomarker for Identifying Cytokeratin 19-Positive Hepatocellular Carcinoma

**DOI:** 10.1155/2023/5424204

**Published:** 2023-02-09

**Authors:** Xiaojun Hu, Qiang Wang, Guobing Huang, Xiang He, Ernesto Sparrelid, Torkel B. Brismar, Yingfang Fan

**Affiliations:** ^1^The Department of General Surgery & Hepatobiliary Surgery, Zhujiang Hospital, Southern Medical University, Guangzhou, China; ^2^Department of Hepatobiliary Surgery, The Fifth Affiliated Hospital of Southern Medical University, Guangzhou, China; ^3^Division of Medical Imaging and Technology, Department of Clinical Science, Intervention and Technology (CLINTEC), Karolinska Institutet, Stockholm, Sweden; ^4^Division of Radiology, Department of Clinical Science, Intervention and Technology (CLINTEC), Karolinska Institutet, Karolinska University Hospital, Stockholm, Sweden; ^5^Division of Surgery, Department of Clinical Science, Intervention and Technology (CLINTEC), Karolinska Institutet, Karolinska University Hospital, Stockholm, Sweden

## Abstract

**Purpose:**

One subtype of hepatocellular carcinoma (HCC), with cytokeratin 19 expression (CK19+), has shown to be more aggressive and has a poor prognosis. However, CK19+ is determined by immunohistochemical examination using a surgically resected specimen. This study is aimed at establishing a radiomics signature based on preoperative gadoxetic acid-enhanced MRI for predicting CK19 status in HCC. *Patients and Methods*. Clinicopathological and imaging data were retrospectively collected from patients who underwent hepatectomy between February 2015 and December 2020. Patients who underwent gadoxetic acid-enhanced MRI and had CK19 results of histopathological examination were included. Radiomics features of the manually segmented lesion during the arterial, portal venous, and hepatobiliary phases were extracted. The 10 most reproducible and robust features at each phase were selected for construction of radiomics signatures, and their performance was evaluated by analyzing the area under the curve (AUC). The goodness of fit of the model was assessed by the Hosmer-Lemeshow test.

**Results:**

A total of 110 patients were included. The incidence of CK19(+) HCC was 17% (19/110). Alpha fetoprotein was the only significant clinicopathological variable different between CK19(-) and CK19(+) groups. A majority of the selected radiomics features were wavelet filter-derived features. The AUCs of the three radiomics signatures based on arterial, portal venous, and hepatobiliary phases were 0.70 (95% CI: 0.56-0.83), 0.83 (95% CI: 0.73-0.92), and 0.89 (95% CI: 0.82-0.96), respectively. The three radiomics signatures were integrated, and the fusion signature yielded an AUC of 0.92 (95% CI: 0.86-0.98) and was used as the final model for CK19(+) prediction. The sensitivity, specificity, accuracy, positive predictive value, and negative predictive value of the fusion signature was 0.84, 0.89, 0.88, 0.62, and 0.96, respectively. The Hosmer-Lemeshow test showed a good fit of the fusion signature (*p* > 0.05).

**Conclusion:**

The established radiomics signature based on preoperative gadoxetic acid-enhanced MRI could be an accurate and potential imaging biomarker for HCC CK19(+) prediction.

## 1. Introduction

Hepatocellular carcinoma (HCC) ranks the seventh most common cancer and is the second leading cause of cancer-related mortality worldwide [1]. The prognosis of HCC remains unfavorable with a 5-year overall survival rate of less than 20% and a recurrence rate of 70% within 5 years after curative surgery [2]. The notable heterogeneity of HCC explains why patients with the same clinical stage, such as that stratified by tumor-node-metastasis system or the Barcelona clinic liver cancer (BCLC) staging system, vary in the prognosis [3]. Knowledge of the histopathological features can guide patient stratification, therapeutic decision-making, and prognosis prediction, providing complementary value to the traditional clinical staging systems [4]. Development of techniques for characterization of HCC prior to treatment might improve clinical outcome [5, 6].

Cytokeratin 19 (CK19) is a keratin-containing intermediate filament protein, which is constantly expressed in epithelial cells [7]. It is a well-established biomarker of hepatic progenitor cells and cholangiocytes that in adults cannot be detected in normal hepatocytes [8, 9]. Previous studies have demonstrated that patients with HCC expressing CK19 (i.e., CK19(+) HCC) have a more severe tumor invasion, a higher rate of lymph node metastasis, earlier recurrence, and worse survival rate compared to those with CK19(-) HCC [10, 11]. Due to the aggressive behavior of CK19(+) HCC, some researchers advocated that this molecular subtype of HCC should be categorized as an independent pathological entity, separating it from other phenotypes displaying features of cholangiocellular differentiation, for instance, cholangiocellular carcinoma and combined HCC-cholangiocarcinoma [9, 10]. However, the CK19(+) status is determined at postoperative histopathological examination. A noninvasive approach based on preoperative examinations would therefore be of great value to shed light on the heterogeneity of HCC.

Gadoxetic acid-enhanced MRI is a commonly used examination in clinical practice. Different from other extracellular contrast media, gadoxetic acid can be absorbed by the hepatocytes and form a specific hepatobiliary phase at approximately 20 min after administration [10]. The superb tissue contrast provided by gadoxetic acid-enhanced MRI improves detection, diagnosis, and characterization of liver lesions [10, 12]. A number of studies have explored an association between radiologic semantic features and CK19(+) HCC, such as arterial rim enhancement, irregular tumor margin, and the Liver Reporting & Data System (LI-RADS) features including nonperipheral washout and targetoid appearance [10, 12]. However, these features are limited by suboptimal reproducibility and insufficient accuracy for CK19(+) status prediction.

In recent years, radiomics has emerged as a burgeoning field that captures a big number of imaging features from clinical, routinely used images and converts them into mineable, quantitative data for modelling [13, 14]. The basic assumption underlying radiomics is that the alterations on molecular/cellular levels are reflected in the images. Radiomics analysis can detect these delicate phenotype changes and quantify them [15]. Through machine learning algorithms, prediction models can be developed for diagnosis, predicting prognosis, and treatment response [16, 17].

**Figure 1:**
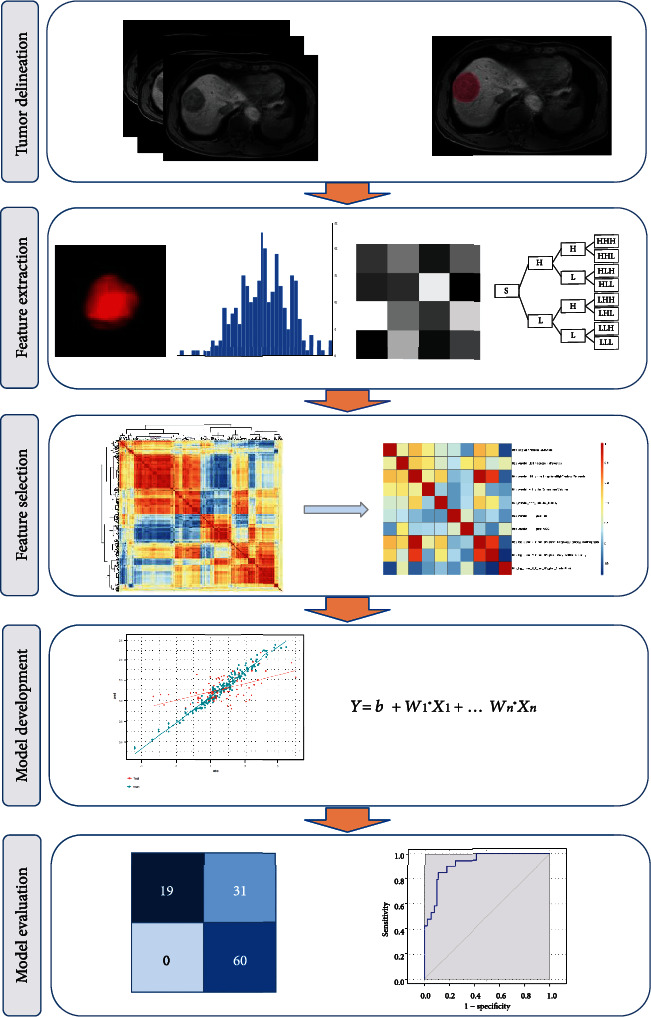
Radiomics workflow for CK19 status prediction in hepatocellular carcinoma. The workflow consists of five key steps. Step 1: after acquisition of preoperative MRI images, tumor segmentation was performed by using ITK-SNAP software. Step 2: radiomics features were extracted from the delineated tumor on the three phases (arterial phase, portal venous phase, and hepatobiliary phase). Step 3: top 10 reproducible and informative features were selected by inter-/intraclass agreement analysis, correlation analysis, and the recursive feature elimination. Step 4: the selected features were subjected to logistic regression analysis to construct the prediction models. Step 5: the model performance was evaluated by the area under the curve.

The aim of this study was to develop a radiomics signature for predicting HCC CK19 status by radiomics analysis on preoperative gadoxetic acid-enhanced MRI images. The development of the radiomics signature in this study followed a “standard radiomics workflow,” which consists of image acquisition, tumor segmentation, feature extraction and selection, model construction, and evaluation, as shown in Figure 1. Besides, the association between clinicopathological variables and HCC CK19 status and the added value of the clinicopathological variable to the radiomics signature were also evaluated.

## 2. Materials and Methods

### 2.1. Study Design and Patient Characteristics

This retrospective study was approved by the Ethics Committee of Zhujiang Hospital, Southern Medical University (No. 2022-KY-027-01). Written informed consent was waived due to the retrospective nature of this research.

**Figure 2:**
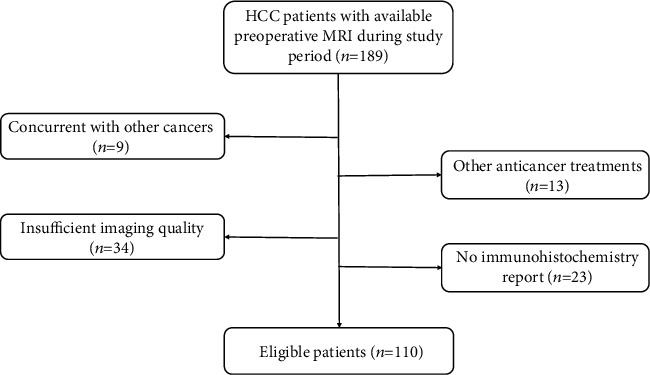
Flowchart of patient selection in this study. Note: HCC: hepatocellular carcinoma; MRI: magnetic resonance imaging.

Patients who underwent a liver resection between February 2015 and December 2020 were identified through the hospital medical information system. The following inclusion criteria were applied: (1) MRI performed within two weeks before surgery; (2) solitary HCC confirmed by postoperatively histopathological examination; and (3) available pathology result of CK19 status. Exclusion criteria were (1) anticancer treatment before liver resection, such as radiofrequency ablation, hepatectomy, or transarterial chemoembolization; (2) other concurrent cancers; and (3) insufficient imaging quality for radiomics analysis, for instance, motion artifact. The following clinical variables were collected: gender, age, hepatitis status, cirrhosis status, serum alpha-fetoprotein (AFP) level, platelet, Child-Pugh score, Model for End-Stage Liver Disease (MELD) score, tumor size, and tumor number. The process of patient selection is described in Figure 2.

### 2.2. Histopathological Examination of CK19

Histopathological examination and diagnosis of CK19 were conducted in line with the World Health Organization criterion [18, 19]. Immunohistochemical staining was carried out on formalin-fixed, paraffin-embedded sections of surgically resected HCC specimens. The monoclonal antibody against CK19 (1 : 200, A53-B/A2.26, Maixin Co., China) was adopted. Immunoreactivity was measured by the percent of areas that were positive for the CK19 marker. CK19 expression was regarded as positive if the percentage of moderate or intense staining tumor cells was 5% or greater [20].

### 2.3. MRI Acquisition

**Figure 3:**
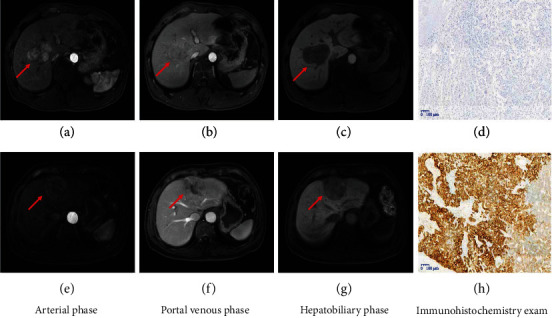
Representative images of different phases of gadoxetic acid-enhanced MRI and CK19 status by immunohistochemical staining (CK19 negative (a–d); CK19 positive (e–h)).

All patients underwent gadoxetic acid-enhanced MRI using a 3.0 T scanner (Ingenia, Philips Healthcare, Best, Netherlands). The imaging series were comprised of a basic series followed by a four-phase postcontrast series with T1 high-resolution isotropic volume examination. Dynamic fat-suppressed T1-weighted 3D images were obtained using turbo field echo (mDixon) in the arterial phase, portal venous phase, delayed phase, and hepatobiliary phase at 18-20 s, 45-50 s, 180 s, and 20 min, respectively, after administration of gadoxetic acid (Primovist, Bayer, Germany). Contrast medium was dosed at 0.025 mmol/kg and injected at a rate of 2.5 mL/s via a power injector. The scanning parameters is provided in Supplemental sTable 1. Arterial phase, portal venous phase, and hepatobiliary phase images were selected for radiomics analysis in this study. A pair of representative cases are shown in Figure 3.

### 2.4. Tumor Segmentation and Inter-/Intraobserver Agreement Evaluation

An open-source software ITK-SNAP (version 3.8.0) was applied to perform tumor segmentation in a manual manner. Initially, imaging data of 20 patients were randomly selected and delineated by two researchers (X.H and G.H). The first researcher (X.H) repeated it one month later. The interobserver and intraobserver agreement was expressed by the interclass correlation coefficient (ICC). The tumor segmentation of the remaining 90 patients was then completed by the same researcher (X.H). The features with ICC > 0.75 were selected for further analysis. When delineating tumors, the researchers had no knowledge about the patients' clinical and histopathological information.

### 2.5. Imaging Preprocessing and Radiomics Feature Extraction

The delineated volume of interest (VOI) of liver tumor was resampled into a voxel size of 1 × 1 × 1 mm^3^ [21] using an interpolation approach, and the intensity histogram of the gray scale was discretized into a width bin of 25. After imaging preprocessing, radiomics features were extracted by the package “pyradiomics” (version 3.0.1, https://github.com/AIM-Harvard/pyradiomics) in Python (version 3.7). The following categories of radiomics features were extracted: (a) shape features, (b) first-order statistics features, (c) gray-level cooccurrence matrix-derived features, (d) gray-level run length matrix-derived features, (e) gray-level size zone-derived features, (f) gray-level dependence matrix-derived features, and (g) neighboring gray tone difference matrix features.

Two filters, wavelet and Laplacian of Gaussian (kernel size: 1, 2, and 3), were applied to transform the original images to obtain more textural information [22]. In total, 1130 features were exacted from each VOI. Detailed information about the radiomics features is available at https://pyradiomics.readthedocs.io/en/latest/features.html. A prefix of “ap,” “pvp,” and “hbp” was artificially added to denote features from different phases.

### 2.6. Feature Selection and Radiomics Signature Development

As the radiomics features were high-dimension data, feature selection was conducted to avoid potential overfitting during modelling. This process was finished by a two-step strategy [23]: first, correlation analysis was applied between a couple of features and a random one was omitted if their correlation coefficient was >0.99. Second, the recursive feature elimination (RFE) algorithm was exploited to rank the features according to their importance in discrimination of CK19 status. RFE is a wrapper-type feature selection strategy and commonly used in machine learning field. It fits a model and then removes less important features recursively until it reaches a predefined number. The features selected by RFE were then subjected to the logistic regression analysis to construct a radiomics signature, and 5-fold cross-validation was performed to avoid the potential model overfitting. To evaluate the integral performance of the radiomics features, a fusion radiomics signature was established by radiomics risk score (namely, “Rad-score”) of the three phases. The Rad-score was determined by a linear combination of the features in each radiomics signature model weighted by their corresponding coefficients.

### 2.7. Statistical Analysis

Categorical variables were presented as count with frequency, and the difference was compared by the chi-square test or Fisher's exact test. To detect independent clinicopathological indicators for CK19(+) prediction, univariate logistic regression analysis was first applied followed by multivariate logistic regression analysis for variables with *p* < 0.05. The area under the receiver operating characteristic (AUC) was used to evaluate the performance of the model. Sensitivity, specificity, accuracy, positive predictive value, and negative predictive value were also determined [24, 25], with optimal cut-off values determined by Youden index, which measures a diagnostic test's capacity to balance sensitivity and specificity by the following formula: sensitivity + specificity − 1 [26]. The Delong test was applied to evaluate the difference between AUCs. The goodness of fit of the model was assessed by the Hosmer-Lemeshow test, with *p* > 0.05 indicating a good consistency. Calibration curve was plotted to evaluate the consistency between the model predicted probability and the actual probability by bootstrapping 2000 times of the samples. The significant level of *p* value was set at 0.05. All statistical analyses and graphs were performed by R software (version 4.0, R Foundation for Statistical Computing, Vienna, Austria).

## 3. Results

### 3.1. Patient Characteristics and the Significant Clinicopathological Indicator

The included 110 patients consisted of 100 males and 10 females, with approximate two-thirds aged less than 60 years (67.3%). CK19(+) HCC was observed in 19 patients (17.3%).

The level of AFP and CA19-9 showed a significant difference between CK19(-) and CK19(+) groups, while the other variables were not significantly different. Detailed baseline and comparison information is provided in Table 1.

Base on the univariate logistic regression analysis, AFP was detected as the only significant variable associated with CK19(+) and the odds ratio value was 5.38 (95% confidence interval (CI) (1.88-15.34), *p* < 0.05) (Table 2). Multivariate regression analysis was abandoned.

### 3.2. Radiomics Feature Selection and Signature Construction

The intraobserver ICC, based on two repeated measurements by the researcher X.H, were 0.84 (0.76-0.90) in the arterial phase, 0.81 (0.75-0.87) in the portal venous phase, and 0.88 (0.82-0.93) in the hepatobiliary phase. The interobserver ICC, between the researchers X. H and G. H, were 0.79 (0.74-0.87) in the arterial phase, 0.77 (0.68-0.84) in the portal venous phase, and 0.82 (0.73-0.89) in the hepatobiliary phase.

Among the 1130 features extracted from each phase, 804 of them (71%) showed an ICC > 0.75. These 804 stable and reproducible features were further filtered by removing one of the paired features with correlation coefficients > 0.99, with 526, 542, and 528 features remaining at the arterial phase, portal venous phase, and hepatobiliary phase, respectively. The top 10 features ranked by the RFE algorithm in each phase were selected for modelling using the classifier of logistic regression analysis. Three radiomics signatures based on the three phases of features, as well as a fusion radiomics signature integrating these three signatures for CK19(+) HCC prediction, were then constructed. The formula of the three radiomics signatures is provided in Supplemental sTable 2, and fusion radiomics signature was:

(1) $$\begin{array}{c} \text{Fusion radiomics signature}=-4.88+3.59*\text{Rad}-\text{score}\left(\text{arterial phase}\right)+6.31*\text{Rad}-\text{score}\left(\text{portal venous phase}\right)+5.48*\text{Rad}-\text{score}\left(\text{hepatobiliary phase}\right). \end{array}$$

### 3.3. Performance of the Radiomics Signature

**Figure 4:**
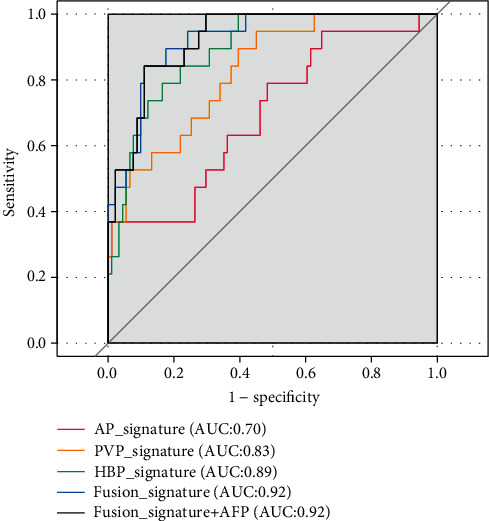
Receiver operating characteristic curves of the different radiomics signatures. Note: AP: arterial phase; PVP: portal venous phase; HBP: hepatobiliary phase; AFP: alpha-fetoprotein; AUC: area under the receiver operating characteristic curve.

**Figure 5:**
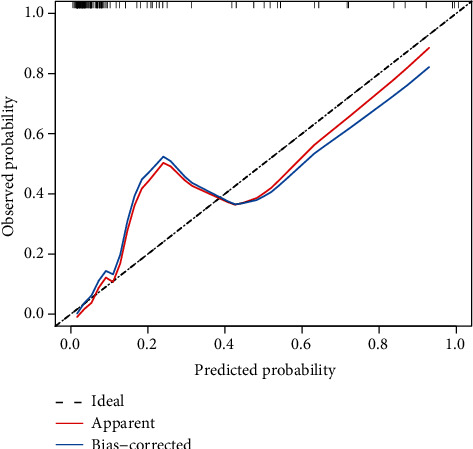
Calibration plot of the fusion radiomics signature in prediction of CK19 status. The probabilities predicted by the fusion radiomics signature (“apparent,” red line) and the bootstrap model (2000 times) (“bias-corrected,” blue line) showed a good consistency. The dashed diagonal represents an “ideal” situation in which the model predicted probability perfectly equals to the observed probability.

The three radiomics signatures based on features from the arterial, portal venous, and hepatobiliary phases yielded an AUC of 0.70 (95% CI: 0.56-0.83), 0.83 (95% CI: 0.73-0.92), and 0.89 (95% CI: 0.82-0.96), respectively. The AUC of the fusion radiomics signature was 0.92 (95% CI: 0.86-0.98) (Figure 4). No further significant improvement of AUC could be observed when incorporating AFP, the only significant clinicopathological variable, into the fusion signature (*p* = 0.82 at the Delong test). To make the prediction model simple, the fusion radiomics signature was therefore chosen as the final model for CK19(+) prediction. The sensitivity, specificity, accuracy, positive predictive value, and negative predictive value of the fusion signature were 0.84, 0.89, 0.88, 0.62, and 0.96, respectively, at a cut-off value of 0.08 (Table 3). The goodness of fit of the fusion radiomics signature was good, as evaluated by the Hosmer-Lemeshow test (*p* > 0.05). Calibration plot showed that the model predicted probability was well consistent with the observed probability (Figure 5).

## 4. Discussion

In the current study, a radiomics signature, which takes advantage of HCC imaging features at the arterial, portal venous, and hepatobiliary phases of preoperative gadoxetic acid-enhanced MRI for prediction of CK19 status, was constructed. The fusion radiomics signature integrates the signatures of three dynamic contrast medium phases and has excellent best prediction performance, indicating that accurate CK19(+) identification can be made preoperatively.

The incidence of CK19(+) HCC in our cohort was 17%, which is in accordance with the reported incidence of 10% to 30% [9]. Several studies have previously explored the role of radiomics in the prediction of CK19 status. In a study with 227 patients, radiomics analysis was carried out on four phases of gadoxetic acid-enhanced MRI (arterial, portal venous, delay, and hepatobiliary phases) for HCC CK19 status prediction [27]. They applied the least absolute shrinkage and selection operator algorithm to select the informative and powerful features. Its fusion radiomics signature (integrating arterial and hepatobiliary phases) yielded an AUC of 0.95 in the training cohort and 0.82 in the test cohort [27]. The performance of our model was similar to this result. Another study adopted T2-weighted images and diffusion-weighted images of gadoxetic acid-enhanced MRI for predicting CK19(+) HCC [28]. The radiomics model was developed by using an artificial neural network based on 257 patients from three medical centers and yielded an AUC of 0.86, 0.73, and 0.79 in one training and two validation cohorts, respectively [18]. That study also compared different classifiers in model development, and artificial neural network seemed a superiority to logistic regression, support vector machine, and random forest [18]. However, that study seemed to be a diagnostic case-control study with a 41% prevalence rate of CK19(+), where the artificially high incidence may introduce case selection bias and overestimation of the model performance [29–31].

Conventional radiological features on gadoxetic acid-enhanced MRI have also been used to predict CK19(+) HCC. Choi et al. identified four independent indicators (irregular tumor margin, arterial rim enhancement, tumor-to-liver signal intensity ratio at the hepatobiliary phase, and tumor-to-liver apparent diffusion coefficient ratio) and found that combination of any three of these four features can obtain an AUC of 0.77 for CK19(+) prediction [10]. Another research also detected arterial rim enhancement as an independent imaging biomarker for CK19(+) identification with an AUC of 0.64 [32]. It seems that the overall performance of these models is inferior to that of the radiomics features. However, the number of such studies is small; therefore, no solid conclusion on the superiority of either of these two approaches could be drawn.

Radiomics features can also be extracted from MR images obtained without contrast medium for CK19 status prediction. In one study, based on susceptibility-weighted imaging, a prediction model with an AUC of 0.91 was achieved [33], similar performance to our model. However, that study was based on a limited sample size with only 53 patients included. To our knowledge, there is no successful radiomics signature based on pretreatment computed tomography (CT) images. Such a model would be of great interest as CT is much more available and reproducible than MRI.

In recent years, a novel subtype of HCC that expresses biomarkers of both HCC (e.g., hepatocyte-1) and cholangiocellular carcinoma (e.g., CK19 or CK7) has been proposed, the so-called “dual-phenotype hepatocellular carcinoma” [34, 35]. Patients with dual-phenotype hepatocellular carcinoma have a poor prognosis and survival rate [34, 36]. In 2019, Huang et al. performed radiomics analysis on gadoxetic acid-enhanced MRI for preoperative diagnosis of dual-phenotype hepatocellular carcinoma [37]. By combining different contrast medium phases and classifiers, a prediction model with an AUC of 0.78 was developed.

In terms of the 30 radiomics features selected for construction of the three signatures, no features overlaid in the three phases. A majority of the features were wavelet derived (23/30, 77%), and only four features were from the original images. The wavelet filter is an effective tool to decompose an image into high- and low-frequency signals along the *x*-, *y*-, and *z*-axes, which effectively removes potential noise and better reflects tumor heterogeneity [38, 39]. In HCC research, wavelet feature-based radiomics have been shown to effectively predict microvascular invasion, tumor grade, and differentiation [40, 41]. Another explanation for the great influence from wavelet derived features is that the number of such features is big, in this study 744/1120 or 66%. This makes them a high probability to remain in the prediction model after dimension reduction. Unfortunately, there is no consensus on terminology or on how to select the highly correlated features. This makes a comparison of radiomics features extracted in different studies impossible.

In this study, AFP was the only significant clinicopathological variable associated with HCC CK19(+). This finding was in line with other CK19(+) studies [12, 27, 32, 42]. AFP is one of the first discovered tumor biomarkers and is widely applied by clinical guidelines for diagnosis of HCC and recurrence surveillance after surgery [43–45]. A large body of studies have demonstrated that elevated AFP levels in HCC patients were correlated with poor tumor differentiation, microvascular invasion, early recurrence, and poor prognosis [46, 47]. This is consistent with the aggressive behavior of CK19(+) HCC. However, the addition of AFP did not show any significant improvement in the prediction performance of the fusion radiomics model.

There are some limitations in this study to be acknowledged when interpreting our findings. First, although the radiomics signature showed a very high predictive performance, it was developed on a limited sample size collected retrospectively in a single center. The generalization of our model still requires external cohorts with a large study population to validate before translation into clinical utility. Second, only two filters (wavelet and Laplacian of Gaussian) were applied in this study, while there are nine filters in the pyradiomics package (https://pyradiomics.readthedocs.io/en/latest/index.html?highlight=wavelet#filter-classes). Future studies can be designed to compare the performance of different filters and their combinations. Third, only RFE algorithm was adopted in this study for model development. A comparison between RFE and other commonly used machine learning algorithms (such as support vector machine, XGBoost) in prediction performance would be useful to select a more powerful algorithm. Based on above-mentioned two points, the discriminative ability of the model with a more powerful algorithm and informative filter(s) can be further improved. Four, although it had the highest AUC value, the fusion radiomics signature was constructed by three phases of features, meaning a heavier workload and more complexity. An ideal model would be a trade-off between accuracy and simplicity. Lastly, a comparison to conventional semantic radiologic features was not conducted as the goal of this study was to develop an objective tool for CK19 status prediction, whereas conventional semantic radiologic features are hampered by their observer variation. A more objective model using deep learning methods might overcome such variability.

## 5. Conclusions

CK19 status of HCC can be predicted preoperatively by applying a radiomics signature on standard clinical gadoxetic acid-enhanced MRI. Although our study showed a very high predictive power, future research with external independent cohorts is required to validate it before translating into clinical implementation.

## Figures and Tables

**Table 1:** Baseline characteristics of the study population.

	All (*n* = 110)	CK19 (-) (*n* = 91)	CK19 (+) (*n* = 19)	*p* value
Gender				0.68
Male	100 (90.9%)	83 (91.2%)	17 (89.5%)	
Female	10 (9.09%)	8 (8.79%)	2 (10.5%)	
Age (years)				0.36
<60	74 (67.3%)	59 (64.8%)	15 (78.9%)	
≥60	36 (32.7%)	32 (35.2%)	4 (21.1%)	
HBV infection				0.47
No	16 (14.5%)	12 (13.2%)	4 (21.1%)	
Yes	94 (85.5%)	79 (86.8%)	15 (78.9%)	
Cirrhosis status				0.60
No	61 (55.5%)	52 (57.1%)	9 (47.4%)	
Yes	49 (44.5%)	39 (42.9%)	10 (52.6%)	
ALBI grade				0.52
Grade1	42 (38.2%)	33 (36.3%)	9 (47.4%)	
Grade2	68 (61.8%)	58 (63.7%)	10 (52.6%)	
MELD score				0.68
<9	100 (90.9%)	83 (91.2%)	17 (89.5%)	
≥9	10 (9.09%)	8 (8.79%)	2 (10.5%)	
Tumor size (cm)				0.50
<5 cm	57 (51.8%)	49 (53.8%)	8 (42.1%)	
≥5 cm	53 (48.2%)	42 (46.2%)	11 (57.9%)	
AST (U/L)				1.000
<40	81 (73.6%)	67 (73.6%)	14 (73.7%)	
≥40	29 (26.4%)	24 (26.4%)	5 (26.3%)	
ALT (U/L)				0.59
<40	78 (70.9%)	66 (72.5%)	12 (63.2%)	
≥40	32 (29.1%)	25 (27.5%)	7 (36.8%)	
PT (second)				0.33
<12.1	20 (18.2%)	15 (16.5%)	5 (26.3%)	
≥12.1	90 (81.8%)	76 (83.5%)	14 (73.7%)	
Platelet (×109/L)				0.36
<125	21 (19.1%)	16 (17.6%)	5 (26.3%)	
≥125	89 (80.9%)	75 (82.4%)	14 (73.7%)	
Serum AFP (ng/mL)				0.002^∗^
<400	76 (69.1%)	69 (75.8%)	7 (36.8%)	
≥400	34 (30.9%)	22 (24.2%)	12 (63.2%)	
Serum CA 19-9 (U/mL)				0.04^∗^
<34	92 (83.6%)	73 (80.2%)	19 (100%)	
≥34	18 (16.4%)	18 (19.8%)	0 (0.00%)	
Serum CEA (ng/mL)				0.69
<5	99 (90.0%)	81 (89.0%)	18 (94.7%)	
≥5	11 (10.0%)	10 (11.0%)	1 (5.26%)	

^∗^ *p* < 0.05. AFP: alpha fetoprotein; ALBI: albumin-bilirubin grade; ALT: alanine aminotransferase; AST: aspartate aminotransferase; CA 19-9: carbohydrate antigen 19-9; CEA: carcinoembryonic antigen; CK19: cytokeratin 19; HBV: hepatitis B virus; MELD score: Model for End-Stage Liver Disease score; OR: odds ratio; PT: prothrombin time.

**Table 2:** Univariate logistic regression analysis of clinicopathological variables associated with CK19 status in hepatocellular carcinoma.

Variable	OR (95% CI)	*p* value
Gender (male vs. female)	1.22 (0.24-6.26)	0.81
Age (<60 vs. ≥60 years)	0.49 (0.15-1.61)	0.24
HBV infection (no vs. yes)	0.57 (0.16-2.01)	0.38
Cirrhosis status (no vs. yes)	1.48 (0.55-3.99)	0.44
ALBI grade (grade 1 vs. grade 2)	0.63 (0.23-1.71)	0.37
MELD score (<9 vs. ≥9)	1.22(0.24-6.26)	0.81
Tumor size (<5 vs. ≥5 cm)	1.60 (0.59-4.36)	0.35
AST (<40 vs. ≥40 U/L)	1.00 (0.32-3.06)	1.00
ALT (<40 vs. ≥40 U/L)	1.54 (0.54-4.36)	0.42
PT (<12.1 vs. ≥12.1 sec)	0.55(0.17-1.77)	0.32
Platelet (<125 vs. ≥125 × 10^9^/L)	0.60 (0.19-1.90)	0.38
Serum AFP (<400 vs. ≥400 ng/mL)	5.38 (1.88-15.34)	<0.001^∗^
Serum CA 19-9 (<34 vs. ≥34 U/mL)	0 (0-Inf)	0.99
Serum CEA (<5 vs. ≥5 ng/mL)	0.45 (0.05-3.74)	0.46

^∗^ *p* < 0.05. AFP: alpha fetoprotein; ALBI: albumin-bilirubin grade; ALT: alanine aminotransferase; AST: aspartate aminotransferase; CA 19-9: carbohydrate antigen 19-9; CEA: carcinoembryonic antigen; CI: confidence interval; HBV: hepatitis B virus; MELD score: Model for End-Stage Liver Disease score; OR: odds ratio; PT: prothrombin time.

**Table 3:** Performance of the established radiomics signature in prediction of CK19 status.

	AUC (95% CI)	Threshold	Accuracy	Sensitivity	Specificity	PPV	NPV
AP radiomics signature	0.70 (0.56-0.83)	0.32	0.88	0.37	0.99	0.88	0.88
PVP radiomics signature	0.83 (0.73-0.92)	0.14	0.65	0.89	0.60	0.32	0.96
HBP radiomics signature	0.89 (0.82-0.96)	0.22	0.83	0.79	0.84	0.50	0.95
Fusion radiomics signature^#^	0.92 (0.86-0.98)	0.19	0.88	0.84	0.89	0.62	0.96
Fusion radiomics signature + AFP	0.92 (0.83-0.97)	0.17	0.88	0.84	0.89	0.62	0.96

AFP: alpha fetoprotein; AP: arterial phase; AUC: area under the receiver operating characteristic curve; CI: confidence interval; HBP: hepatobiliary phase; NPV: negative predictive value; PPV: positive predictive value; PVP: portal venous phase. ^#^Radiomics model consists of three radiomics scores derived from AP-, PVP-, and HBP-based model.

## Data Availability

The datasets used and/or analyzed during the current study are available from the corresponding author upon reasonable request.

## Supplementary Materials

Supplementary Materials sTable 1: gadoxetic acid-enhanced MRI scanning parameters. sTable 2: formulas of the radiomics models based on different phases.
