# Supplementary material for: Gadoxetic Acid-Enhanced MRI-Based Radiomics Signature: A Potential Imaging Biomarker for Identifying Cytokeratin 19-Positive Hepatocellular Carcinoma
Source: Comput Math Methods Med. 2023 Feb 9;2023:5424204. doi: 10.1155/2023/5424204 (PMC9940957; doi:10.1155/2023/5424204)
Supplement: Supplementary Materials — sTable 1: gadoxetic acid-enhanced MRI scanning parameters. sTable 2: formulas of the radiomics models based on different phases. [file 5424204.f1.docx]

**Supplementary materials**

Gadoxetic acid-enhanced MRI based radiomics signature: A potential imaging biomarker for identifying cytokeratin 19 positive hepatocellular carcinoma

**sTable 1. Gadoxetic acid enhanced MRI scanning parameters**

| Phase | TR (ms) | TE (ms) | FOV (mm*mm) | Flip angle (degree) | Slice thickness (mm) | Matrix size (mm*mm) |
| --- | --- | --- | --- | --- | --- | --- |
| AP | 3.7 | 1.31 | 330*241 | 10 | 2.5 | 220*160 |
| PVP | 3.2 | 1.13 | 240*330 | 10 | 2.5 | 136*182 |
| DP | 3.2 | 1.13 | 240*330 | 10 | 2.5 | 136*182 |
| HBP | 3.2 | 1.13 | 240*330 | 10 | 3.5 | 172*183 |

*Note: AP, arterial phase; DP, delayed phase; FOV, field of view; HBP, hepatobiliary phase; PVP, portal venous phase; TE, echo time; TR, repetition time.*

**sTable 2. Formulas of the radiomics models based on different phases.**

**2.1** Radiomics signature based on **arterial phase**:

-1.682+0.358*ap_log-sigma-3-0-mm-3D_glcm_Idmn-

0.212* ap_wavelet-HHH_glszm_LargeAreaEmphasis+

0.306* ap_wavelet-HHL_ngtdm_Contrast-

0.278* ap_wavelet-HHL_ngtdm_Busyness-0.487* ap_wavelet-HHL_glcm_Idmn-

0.238* ap_wavelet-HHL_glszm_SmallAreaEmphasis+

0.14* ap_wavelet-HLL_glszm_LargeAreaLowGrayLevelEmphasis+

0.509* ap_wavelet-LHH_glcm_Imc2+0.488* ap_wavelet-LLH_ngtdm_Busyness+

0.295* ap_original_glszm_LargeAreaEmphasis

**2.2** Radiomics signature based on **portal venous phase**:

-1.884-0.534*pvp_log-sigma-3-0-mm-3D_gldm_DependenceNonUniformityNormalized+

0.515* pvp_log-sigma-3-0-mm-3D_glszm_SmallAreaLowGrayLevelEmphasis+

0.407* pvp_wavelet-HHL_ngtdm_Contrast+

0.215* pvp_wavelet-HHL_glszm_SmallAreaEmphasis+

0.423* pvp_wavelet-LHH_ngtdm_Busyness+

0.684* pvp_wavelet-LLH_glrlm_LongRunLowGrayLevelEmphasis+

0.108* pvp_wavelet-LLH_ngtdm_Busyness+

0.529* pvp_wavelet-LLH_glszm_LowGrayLevelZoneEmphasis+

0.912* pvp_original_glcm_Imc2-0.805* pvp_original_gldm_LowGrayLevelEmphasis

**2.3** Radiomics signature based on **hepatobiliary phase**:

-1.990 -1.122* hbp_log-sigma-3-0-mm-3D_ngtdm_Strength+

0.552* hbp_log-sigma-3-0-mm-3D_glszm_SmallAreaLowGrayLevelEmphasis+

0.304* hbp_log-sigma-2-0-mm-3D_glcm_MCC+

0.48* hbp_log-sigma-2-0-mm-3D_glcm_Correlation-0.471* hbp_wavelet-HHL_glcm_InverseVariance-

0.41* hbp_wavelet-HLL_glcm_Imc1+ 0.363* hbp_wavelet-HLL_glcm_Correlation+

0.245* hbp_wavelet-HLL_glszm_LargeAreaLowGrayLevelEmphasis+

0.338* hbp_wavelet-LLH_ngtdm_Busyness+0.284* hbp_original_ngtdm_Busyness
